# The perceived impact of artificial intelligence on academic learning

**DOI:** 10.3389/frai.2025.1611183

**Published:** 2025-10-03

**Authors:** Mariana Dogaru, Olivia Pisică, Cosmin-Ștefan Popa, Andrei-Adrian Răgman, Ilinca-Roxana Tololoi

**Affiliations:** ^1^Department for Teachers Initial Training, National University of Science and Technology POLITEHNICA Bucharest, Bucharest, Romania; ^2^Computer Science Department, Faculty of Automatic Control and Computers, National University of Science and Technology POLITEHNICA Bucharest, Bucharest, Romania

**Keywords:** ChatGPT, education, competences, AI literacy, AI support in academic performance, personalized learning

## Abstract

Generative artificial intelligence, such as ChatGPT, is transforming higher education by enabling personalized learning, while raising ethical challenges. This study explores how technical university students perceive and leverage ChatGPT in academic tasks, focusing on motivation, learning outcomes, and ethical awareness. Using the Technology Acceptance Model and Self-Determination Theory, the research surveyed 84 students from a technical university via a 5-point Likert-scale questionnaire. Six salient dimensions of student engagement with ChatGPT emerged: perceived usefulness for problem solving, learning retention and skill acquisition, structured interaction with familiar content, consultation on unfamiliar topics, preference for conciseness, and confidence in the accuracy of AI responses. Students who perceived ChatGPT as a valuable resource for addressing academic problems reported enhanced motivation and competence, and frequent structured interaction was linked to the practice of verifying uncertain information, indicating the emergence of AI literacy. However, extensive reliance was correlated with dependence and limited citation practices, revealing risks to academic integrity. By examining ChatGPT’s role in STEM education, this study substantiates the relevance of AI literacy training and institutional policies to ensure responsible use. The findings offer practical insights for educators to integrate AI tools effectively while fostering critical thinking and academic integrity in technology-driven learning environments.

## 1 Introduction

The launch of ChatGPT, created by OpenAI, has transformed educational technology by introducing a dynamic, dialog-driven approach to learning that surpasses conventional tools like textbooks and video courses (Hosseini et al., 2023). Its capacity to provide immediate, tailored explanations and generate diverse content has generated both excitement and unease within academic circles (Salih et al., 2024). While ChatGPT enhances accessibility and personalizes learning, a concern for plagiarism, over-reliance, and ethical implications have fueled discussions about its responsible integration into education (Nemorin, 2024; Liu and Yushchik, 2024).

This study investigates how technical students perceive and employ ChatGPT in their academic endeavors, evaluating its benefits, potential risks, and effects on learning outcomes. By examining students’ behaviors and perspectives, we address academic concerns regarding AI’s influence and contribute to developing pedagogical approaches that encourage ethical use. Using a questionnaire, we explored how students assess ChatGPT’s usefulness and ease of use, and how these factors influence their decision to adopt it for learning while also identifying ethical challenges they raised (Lai et al., 2023). Our research highlights ChatGPT’s role in facilitating learning, underscores the impact of perceived usefulness and user-friendliness on its adoption, and draws attention to ethical considerations that must be addressed to ensure its responsible use in academic settings (Lai et al., 2023).

## 2 Educational benefits and challenges of ChatGPT

The integration of ChatGPT into higher education has reshaped teaching and learning, sparking extensive research into its pedagogical, ethical, and social implications. This study synthesizes research on the benefits and challenges of ChatGPT, the need for AI literacy, and the role of educators, emphasizing their relevance to our objective: understanding how technical students perceive ChatGPT and the associated risks. These studies inform our questionnaire, which explores usage patterns, ethical awareness, and learning outcomes, addressing gaps in student-centered research within technical disciplines. ChatGPT offers significant benefits for students, particularly in technical fields, by providing personalized, immediate feedback (Egunjobi and Adeyeye, 2024; Salih et al., 2024; Jansen et al., 2024). Its capacity to clarify complex concepts, solve technical problems, and generate texts enhances academic productivity (Segbenya et al., 2024). Chiu (2023) underscores its role in fostering engagement through interactive dialogs, while Sharples (2023) highlights its potential for collaborative learning. These advantages are central to our study, as they are likely to shape students’ perceptions of ChatGPT’s utility, assessed through items such as *“I found ChatGPT helpful for university when…”* (items I1-I5). However, Heeg and Avraamidou (2023) caution that ChatGPT’s effectiveness depends on contextual factors, including prompt quality, suggesting variability in student experiences—an aspect our research seeks to investigate.

The integration of artificial intelligence (AI) into education has generated considerable interest, offering transformative opportunities alongside complex challenges. Tools like ChatGPT, developed by OpenAI, exemplify AI’s potential to redefine academic learning through interactive, individualized support (Hosseini et al., 2023). This section synthesizes the literature on AI’s role in education, focusing on three key dimensions: customized learning, AI literacy, and ethical considerations. These themes are critical to understanding how technical university students perceive and engage with ChatGPT, as explored in this study. The discussion draws on the Technology Acceptance Model (TAM) (Davis, 1989), which links perceived usefulness and ease of use to technology adoption, and Self-Determination Theory (SDT) (Ryan and Deci, 2000), which highlights the role of intrinsic motivation in learning processes.

### 2.1 Personalized learning

AI tools like ChatGPT, automated writing evaluation (AWE) systems, and platforms such as GRAD-AI have changed personalized learning by providing customized feedback, which means adjusting content to fit each student’s unique needs (Egunjobi and Adeyeye, 2024; Yang et al., 2023; Gambo et al., 2024a; Ayman et al., 2023). For instance, ChatGPT supports students in technical disciplines by generating explanations, summarizing texts, and solving problems, thereby enhancing engagement and comprehension (Salih et al., 2024). GRAD-AI, designed for programming courses, automates code grading and provides detailed feedback, reducing instructors’ workload while improving learning efficiency (Gambo et al., 2024b). Similarly, AWE tools like Pigai offer contextualized feedback on English writing, enabling students to refine their skills iteratively (Shi and Aryadoust, 2024).

The flexibility of AI tools, available all the time, addresses diverse learning schedules, particularly for technical students juggling complex coursework (Salih et al., 2024). Egunjobi and Adeyeye (2024) argue that AI’s ability to personalize content fosters intrinsic motivation, aligning with SDT’s emphasis on autonomy and competence. However, Crawford et al. (2024) caution that overreliance on AI may diminish critical thinking if students prioritize quick solutions over profound understanding. Liu and Yushchik (2024) advocate for a balanced approach, integrating AI with traditional pedagogical methods to maintain intellectual rigor. In contrast, Heeg and Avraamidou (2023) highlight AI’s potential to enhance science education by simulating experiments, suggesting that personalization extends beyond text-based support to interactive applications.

Our study investigates how ChatGPT’s personalization features influence technical students’ academic performance and motivation, testing TAM’s premise that perceived usefulness drives adoption. By examining contexts like assignment completion and concept understanding, the study explores whether AI’s tailored support aligns with students’ learning needs or fosters dependency.

In the context of artificial intelligence (AI) utilization, the concepts of “dependency” or “reliance” refer to the degree to which individuals, organizations, or society depend on AI systems for decision-making, task execution, or problem-solving. These concepts encompass both positive and negative dimensions, contingent upon the context. Positive dependency on AI is evident in the efficiency and automation of repetitive tasks, such as data analysis and logistics management, support in decision-making through data-driven recommendations, and enhanced accessibility via virtual assistants. Conversely, negative dependency manifests as a loss of autonomy, characterized by a diminished capacity for independent decision-making and critical thinking, particularly when AI recommendations are accepted without scrutiny or analysis (for instance, an individual might follow an AI-suggested travel route despite knowing its limitations, accepting it without discernment, even if it contains errors). Additionally, over-reliance on AI can render individuals helpless in the event of system failures, such as when a navigation application malfunctions, raising questions about their ability to navigate independently. Such negative dependencies may, over time, lead to the atrophy of cognitive functions, as individuals become increasingly reliant on AI for tasks like time management, potentially losing the ability to perform tasks independently. Ultimately, dependency on AI represents a delicate balance between the benefits of automation and the risks of excessive reliance, highlighting the need for a nuanced approach to integrating AI into daily life and professional practices (Huang et al., 2024; Morales-García et al., 2024; Zhai et al., 2024; Zhang and Xu, 2024; Zhang et al., 2024).

Personalization of learning is not an end in itself, possessing a less sustainable character, whereas Learning Motivation represents the driving force that initiates and redefines the entire intellectual conduct of a student in training for integration into a continuously evolving society that constantly updates the knowledge to be acquired, irrespective of personalized learning. In this context, Learning Motivation supports students in addressing societal challenges, with its expression, understanding, and identification being far more valuable.

Learning motivation, encompassing intrinsic and extrinsic drivers of engagement, offers a more comprehensive and actionable framework, aligns educational content with students’ interests, fostering intrinsic motivation—the desire to learn for its own sake. Therefore, we consider that Learning Motivation, as a construct, better captures the empowering potential of AI-driven learning personalization.

### 2.2 AI literacy

AI literacy, defined as the ability to understand, use, and critically evaluate AI technologies, is increasingly vital in higher education (Tzirides et al., 2024; Yim, 2024). This encompasses technical knowledge (e.g., how large language models function), critical assessment of outputs for accuracy and bias, and awareness of ethical implications (Costa et al., 2024; Nemorin, 2024). For instance, students using ChatGPT must verify responses to avoid misinformation, a concern raised by Hsu and Thompson (2023). Tzirides et al. (2024) propose combining AI tools with human-centered pedagogies to foster literacy, emphasizing skills like problem-solving and digital navigation.

Chiu (2023) argues that AI literacy empowers students to engage with tools like ChatGPT responsibly, enhancing their competence—a core SDT construct. However, Yim (2024) notes that current curricula often lack structured AI literacy programs, leaving students to develop these skills informally. This gap is particularly relevant for technical students, who may overestimate AI’s reliability due to their shallow formal output. The importance of familiarity with technology is highlighted by Segbenya et al. (2024). Nemorin (2024) adds a critical perspective; in order to guarantee equitable literacy development, educators are urged to address Eurocentric biases in AI ethics and integrate diverse cultural perspectives. Comparatively, Lee et al. (2024) and Gouseti et al. (2024) emphasize teacher training as a prerequisite for fostering student literacy, arguing that educators must model critical AI use. Sharples (2023) suggests collaborative workshops where students and teachers experiment with AI tools, promoting shared learning. Our study looks at whether technical students are fully aware of the metacognitive consequences reflected in day-to-day life using AI models (e.g., ChatGPT), especially in checking results and citing sources, to see if they are prepared to use AI responsibly.

### 2.3 Ethical and academic challenges

The adoption of AI in education raises significant ethical challenges, including academic integrity, data privacy, and equitable access (Salih et al., 2024; Ho et al., 2024; Skowronek et al., 2021). ChatGPT’s ability to generate essays and solve problems increases plagiarism risks, as students may submit AI-generated content without proper citation (Salih et al., 2024). Ho et al. (2024) highlight data security concerns, noting that AI platforms collect sensitive student information, necessitating robust privacy policies. Skowronek et al. (2021) warn that unequal access to AI tools can exacerbate educational inequities, particularly in under-resourced institutions.

Crawford et al. (2024) argue that excessive AI use may reduce social interactions, weakening students’ sense of belonging—a critical factor in SDT’s relatedness component. Algorithmic biases, embedded in AI models, can perpetuate stereotypes or inaccuracies, requiring critical scrutiny (Ho et al., 2024; Nemorin, 2024). For example, Thaker and Thaker (2024) notes that biased AI outputs in educational contexts may misguide students, undermining learning outcomes. Parker (2024) and Liu and Yushchik (2024) advocate AI as a supportive tool, not a replacement for educators, who must guide students in ethical practices.

In contrast, Rahiman and Kodikal (2024) highlight AI’s potential for collaborative learning, suggesting that ethical concerns can be mitigated through structured integration. Gouseti et al. (2024) propose explicit ethical frameworks for K-12 and higher education, emphasizing transparency in AI use. Our study looks at how aware students are of ethical issues, like whether they check or reference what ChatGPT produces, to see how their behavior matches those concerns and to help shape teaching methods.

### 2.4 Educators’ evolving roles

Educators must adapt to AI-integrated education, shifting from knowledge providers to facilitators (Guan et al., 2024). Yang et al. (2023) and Shi and Aryadoust (2024) stress training in AI tools to guide students effectively. Ethical training is crucial, ensuring educators model responsible use (Nemorin, 2024). These insights inform our study’s exploration of students’ reliance on ChatGPT versus traditional resources.

### 2.5 Synthesis and research gaps

The literature on ChatGPT and AI in education encompasses a broad range of topics, each contributing to our understanding of its implications for teaching and learning, particularly in technical disciplines. Research highlights ChatGPT’s transformative role in academic practices, emphasizing its capacity to support learning, research, and institutional adoption (Salih et al., 2024). Educators’ perceptions of generative AI’s impact on pedagogy indicate that it requires institutional support and training to maximize its benefits (Lee et al., 2024). In programming education, AI-powered tools like GRAD-AI automate code grading, offering rapid feedback that enhances student learning (Gambo et al., 2024a; Gambo et al., 2024b). AI literacy is emerging as a critical focus, with studies emphasizing the importance of humans developing technological skills to understand AI functionality and integrate it into daily life more quickly. The focus is on centered pedagogical strategies designed to equip students with both technical and ethical skills (Tzirides et al., 2024). The constructive collaboration between AI and augmented reality (AR) is explored for its potential to create personalized, interactive learning experiences, though its relevance to ChatGPT remains secondary (Egunjobi and Adeyeye, 2024; Maphosa and Maphosa, 2023; Saraswat, 2024). AI-based feedback systems, such as those for EFL writing, demonstrate efficacy but require contextualized guidance to be effective (Yang et al., 2023; Shi and Aryadoust, 2024). In science education, AI applications improve teaching, yet limitations in current research indicate that they require further exploration (Heeg and Avraamidou, 2023). Ethical considerations are central, with studies addressing AI’s benefits, risks, and the need for balanced integration to avoid replacing human educators (Thaker and Thaker, 2024; Liu and Yushchik, 2024; Zaman, 2023; Yao and Wang, 2024). Nemorin (2024) advocates for inclusive perspectives in educational contexts, challenging Eurocentric frameworks and underlining collaboration between specialists (technicians, educators, cultural experts etc.) to have an” inclusive” algorithm for AI generating inclusive answers to specific issues. Costa et al., 2024; Pradana et al., 2023 emphasize the critical use of ChatGPT to foster students’ analytical skills and ensure responsible engagement. This emphasizes the importance of ensuring responsible engagement (Costa et al., 2024; Pradana et al., 2023). AI significantly influences academic productivity by supporting research and data analysis; however, challenges such as accessibility continue to persist (Segbenya et al., 2024; Grájeda et al., 2023). Social generative AI holds promise for collaborative learning, but ethical concerns remain (Sharples, 2023; Rahiman and Kodikal, 2024). Broader research directions for AI in education call for interdisciplinary collaboration to address emerging challenges (Hwang et al., 2020). The social and emotional impacts of AI, including perceptions of emotional AI and risks of reduced student belonging, are also critical (Ho et al., 2024; Crawford et al., 2024). Preparing pre-service teachers for AI-integrated education is vital, focusing on their perceptions and identity shifts (Guan et al., 2024; Parker, 2024). Emerging technologies like the Metaverse, while tangential, suggest synergies with generative AI (Qian et al., 2023). AI literacy frameworks for younger learners offer recommendations for higher education, emphasizing societal implications (Yim, 2024). Ethical challenges in K-12 education, such as the need for professional training, are relevant to higher education contexts (Gouseti et al., 2024). Finally, the wider effects of generative AI on teaching show how important it is to build critical skills (Chiu, 2023), and when students interact with automated feedback, it can improve their learning results (Koltovskaia, 2020; Saraswat, 2024). Furthermore, we have listed the main aspects and key conclusions from the literature review below. In comparison to our theme, we aim to emphasize the students’ enthusiastic perception of using AI in the learning process, as shown in Table 1.

**Table 1:** Main aspects of AI in education.

Main aspect	Key conclusions and authors
Learning motivation	AI in education personalizes learning, enhances engagement, and provides rapid feedback (Egunjobi and Adeyeye, 2024; Salih et al., 2024; Escalante et al., 2023; Gambo et al., 2024a).
AI literacy	Understanding AI concepts, critical evaluation, and ethical awareness is essential (Tzirides et al., 2024; Yim, 2024; Costa et al., 2024; Nemorin, 2024).
Ethical challenges	Researchers have identified risks such as plagiarism, data privacy concerns, bias, and decreased social interaction (Salih et al., 2024; Ho et al., 2024; Crawford et al., 2024; Thaker and Thaker, 2024).

## 3 Research methodology

In order to reach our main goal, the methodology applied for the research was rigorous, using certified approaches to achieve the study’s objectives and evaluate the hypotheses. It concerns the design, organization, and execution of the study, with a focus on the methodologies’ intuition and the processes for gathering and analyzing data. This comprehensive explanation guarantees openness and supports the validity of the study. The methodological choices are also discussed to guarantee that the design, analytical techniques, and research objectives are all consistent, demonstrating adherence to the study’s theoretical framework and promoting repeatability in future investigations.

### 3.1 Aim and hypotheses

This study aims to evaluate the perceptions and usage patterns of ChatGPT among technical students, assessing its benefits for academic tasks and risks such as plagiarism and dependency. Drawing on TAM (Lai et al., 2023), we propose the hypotheses found in Table 2.

**Table 2:** Hypotheses for the study in relation to the studied aspects in literature (Table 1).

Code	Hypothesis	Main Aspect
H1	Students who perceive ChatGPT as a valuable tool for problem solving tend to demonstrate stronger learning retention and skill acquisition.	Learning motivation
H2	Students who engage in more structured interactions with familiar topics are more likely to verify the accuracy of ChatGPT-generated output.	AI Literacy
H3	Students who engage more frequently in consulting unfamiliar topics and interacting with familiar ones in a structured way tend to report greater learning gains and increased reliance on ChatGPT.	AI Literacy
H4	Students who often consult unfamiliar topics but engage less in verifying ChatGPT’s output may exhibit behaviors associated with plagiarism.	Plagiarism

### 3.2 Participants and sampling

**Figure 1:**
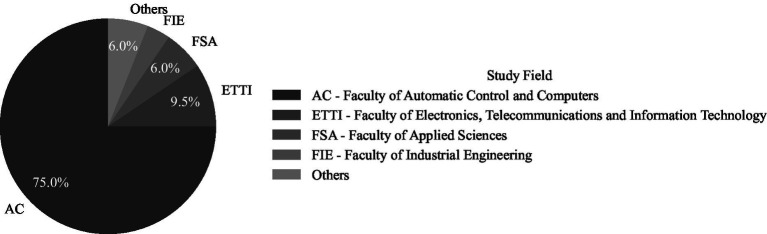
Distribution of respondents by faculty of origin.

The sample is comprised of 84 students from technical domains, selected via **c**onvenience sampling based on their familiarity with technology (Hosseini et al., 2023). Approximately 90% of the students were undergraduates (70% of undergraduates were first year), and 10% of them were master’s students, with a balanced overall gender ratio (approximately 40% of the sampled students were females, and the rest of 60% were males). All participants were students at the National University of Science and Technology Politechnica of Bucharest. The majority (75%) of participants were students from the Faculty of Automatic Control and Computers. The remaining participants follow a study program at the Faculty of Electronics, Telecommunications, and Information Technology (9.5%), the Faculty of Applied Sciences (6%), the Faculty of Industrial Engineering (3.5%), and various other faculties (6%) (see Figure 1). We ensured informed consent from participants and anonymity in compliance to ethical guidelines (Ho et al., 2024).

### 3.3 Research design and instrument

A Google Forms questionnaire, informed by Salih et al. (2024) and Chiu (2023), assessed three theoretical constructs: Learning Motivation (e.g., I37, measuring encouragement), AI Literacy (e.g., I6-8 “*I consult ChatGPT about a topic I know well.”*), and Plagiarism and Ethical Awareness (e.g., I17-19, measuring the level of ChatGPT-generated content integrated in assignments), as denoted in Addendum one. These constructs were defined *a priori* based on established literature and serve a purely organizational role, helping to conceptually structure the survey and guide hypothesis testing. The individual items were chosen carefully such that they can reflect many perspectives on the same subject. For instance, it assesses the level of skepticism toward the generated output, while also examining the scope and quantity of the content produced. Additionally, certain items were designed to evaluate the perceived verbosity of ChatGPT’s responses and the actual capability of the model for summarization. The 38 items were evaluated on a 5-point Likert scale, from 1 (strongly disagree) to 5 (strongly agree). The questionnaire addressed a variety of ChatGPT applications, including text production, problem-solving, and conceptual understanding. To ensure the relevance and content validity of the questionnaire items, a panel of three anonymous university professors—recognized experts in the field—was consulted. Each expert independently rated the items based on their relevance using a 4-point Likert scale. Content Validity Index (CVI) values were then computed following the methodology recommended by Yusoff (2019). Polit and Beck (2006) and Polit et al. (2007) suggest that, for a panel of three experts, a CVI of 1.00 is required to establish item-level validity. The Scale-Level Content Validity Index (S-CVI/Ave), calculated as the average of the Item-Level Content Validity Indices (I-CVIs), was 0.991, indicating a high degree of agreement among the experts. Furthermore, the Scale-Level CVI based on universal agreement among experts (S-CVI/UA) was 0.973, further supporting the overall content validity of the instrument. We shared the questionnaire via university portals for over 2 weeks in March 2024, ensuring anonymity (Ho et al., 2024).

### 3.4 Statistical analysis

We employed descriptive and inferential statistical techniques to examine the implications of the collected data. The descriptive statistics (e.g., medians) present the tendency of the population in question (Dancey and Reidy, 2004; Norušis, 2011). Non-parametric tests such as the Wilcoxon Signed-Rank and the Friedman test for two, respectively more, matched samples were applied where needed because of the ordinal character of Likert-scale data. For the Friedman test, post-hoc analysis (homogeneous subsets and pairwise analysis) was concluded using the Wilcoxon Signed Test adjusted with the Bonferroni correction. To uncover underlying patterns of student interaction with ChatGPT, we conducted Exploratory Factor Analysis (EFA) using Principal Axis Factoring with Promax rotation. The analysis was preceded by checks for sampling adequacy and data suitability. To assess the relationships between factors, a two-tailed Pearson correlation analysis was conducted. A level of significance of α = 0.05 was established before testing the hypotheses, where it is not specified otherwise. The Python 3.11 modules matplotlib, pandas, seaborn, and SciPy were used to construct visualizations for the numerical analysis, which were conducted in SPSS version 26.0.0 (Dancey and Reidy, 2004).

## 4 Results and discussion

An exploratory factor analysis (EFA) was conducted to examine the underlying structure of students’ interactions with ChatGPT in a university context. An iterative trimming procedure was applied to ensure a clean and stable factor structure. The procedure involved three iterations. In the first two iterations of the procedure, items were eliminated based on specific criteria: those with low communalities (below 0.40), low factor loadings (below 0.30), and cross-loadings where the difference between primary and secondary loadings was less than 0.20 were removed. In the third and final iteration, the factor structure was stable, with all retained items exhibiting primary loadings above 0.40 and cross-loading differences of at least 0.20, as shown in Table 3. All communalities exceeded 0.40, suggesting that each item shared a reasonable proportion of variance with the extracted factors. The Kaiser-Meyer-Olkin measure of sampling adequacy (KMO = 0.705) indicated that the data were suitable for factor analysis, and Bartlett’s test of sphericity [*χ*^2^(171) = 517.632, *p* < 0.001] confirmed sufficient correlations among the items. While the exploratory analysis initially yielded seven factors, one factor was removed due to poor reliability (Cronbach’s *α* = 0.464), resulting in a final solution of six interpretable factors.

**Table 3:** Rotated factor table with loadings.

Item	F1	F2	F3	F4	F5	F6	F7
I4	**0.804**	0.201	0.038	0.000	0.095	−0.165	0.183
I3	**0.729**	−0.002	−0.114	0.000	−0.081	0.062	0.091
I30	**0.724**	−0.110	0.121	0.061	−0.023	−0.160	0.002
I1	**0.594**	−0.221	0.028	−0.144	0.143	0.222	−0.296
I23	**0.473**	0.177	−0.147	0.206	−0.117	0.102	−0.037
I22	−0.194	**0.869**	0.032	−0.005	−0.059	−0.063	−0.196
I21	0.141	**0.669**	0.023	−0.023	0.144	−0.029	−0.093
I31	0.120	**0.462**	0.089	−0.022	−0.013	0.108	0.194
I7	0.042	0.055	**0.874**	0.071	−0.002	0.091	0.037
I6	−0.035	0.047	**0.749**	−0.066	−0.055	0.103	−0.033
I24	0.068	−0.125	0.135	**0.846**	0.041	−0.153	−0.134
I34	−0.003	0.044	−0.120	**0.544**	0.036	0.207	0.168
I38	0.018	0.071	−0.017	**0.458**	0.075	0.272	−0.012
I35	−0.169	0.036	−0.076	0.040	**0.880**	0.071	0.038
I36	0.150	0.025	0.007	0.066	**0.586**	−0.118	−0.042
I12	−0.152	−0.055	0.159	0.112	−0.034	**0.826**	0.074
I2	0.276	0.133	0.020	−0.039	−0.043	**0.428**	−0.113
I29	0.076	−0.110	−0.075	0.085	−0.057	0.062	0.688
I10	0.022	−0.059	0.182	−0.172	0.123	−0.042	0.439

Bold values denote the highest loadings associated with each factor in the table. Factor 7 was removed because its loadings were insufficient.

The total variance explained by the six retained factors indicates that the extracted dimensions captured a substantial proportion of the variance in the items. The cumulative variance explained was 52.8% after rotation. This demonstrates that the retained factors collectively represent over half of the variability in students’ responses, reflecting a meaningful multidimensional structure.

In interpreting the six emergent dimensions (Table 4), we discern a rich cognitive–behavioral framework that anchors student interaction with ChatGPT across pragmatic utility, epistemic vigilance, ethical tension, and learning outcomes. The first factor, labeled *Perceived Utility for Problem Solving* ( α = 0.802), reflects students’ perceptions that ChatGPT facilitates the resolution of university-related problems, particularly when addressing technical questions, completing homework, or managing time constraints. The second factor, *Learning Retention and Skill Acquisition* ( α = 0.688), captures the extent to which interacting with ChatGPT enhances understanding, promotes skill retention, and allows students to solve similar problems independently in the future. The third factor, *Structured Interaction with Known Topics* ( α = 0.845), represents consultations with ChatGPT about topics with which students are already familiar, emphasizing the refinement of knowledge through precise instructions or detailed dialog rather than learning new content. The fourth factor, *Consultation on Unfamiliar Topics* ( α = 0.622), encompasses students’ use of ChatGPT to explore and understand topics that are unfamiliar to them, including interpreting complex texts or technical information. The fifth factor, *Conciseness Preference* ( α = 0.619), describes students’ preferences for concise explanations and the challenges posed by verbose responses. The sixth factor, *Verification Behavior* ( α = 0.577), reflects strategies for evaluating the accuracy of ChatGPT responses, such as requesting detailed explanations when uncertain.

**Table 4:** Factors and core items.

Factor code	Factor name	Cronbach’s α	Core items (label: item stem…)
F1	Perceived utility for problem solving	0.802	I4: “Helpful for technical problems” (0.804)
			I3: “Helpful for homework” (0.729)
			I30: “Reach solution faster” (0.724)
			I1: “Helpful when short on time” (0.594)
			I23: “Turn to ChatGPT for unsolved problems” (0.473)
F2	Learning retention and skill acquisition	0.688	I22: “Understand problem approach, not just solution”(0.869)
			I21: “Gained lasting skills from assignments” (0.669)
			I31: “Can handle problem on my own later” (0.462)
F3	Structured interaction with known topics	0.845	I7: “Ask for precise instructions” (0.874)
			I6: “Engage in detailed dialogue” (0.749)
F4	Consultation on unfamiliar topics	0.662	I24: “Consult ChatGPT on unfamiliar topics” (0.846)
			I34: “Ask for explanations of texts/articles” (0.544)
			I38: “Request precise technical info” (0.458)
F5	Conciseness preference	0.619	I35: “Too many words, hard to follow” (0.880)
			I36: “Ask ChatGPT to be concise” (0.586)
F6	Verification behavior	0.577	I12: “Ask to explain in detail if unsure” (0.826)
			I2: “Helpful for understanding concepts” (0.428)

All retained items demonstrated clear factor membership, with primary loadings above 0.40 and sufficient differences from secondary loadings. A correlation matrix (Table 5) confirmed that the extracted factors were conceptually distinct, with inter-factor correlations ranging from −0.14 to 0.53. The three-iteration EFA procedure yielded a clean and stable factor structure, providing six reliable factors that capture multiple dimensions of students’ interactions with ChatGPT, including immediate utility, learning outcomes, structured engagement, exploratory usage, preferences for conciseness, and verification behaviors.

**Table 5:** Pearson correlation between factors.

Factor	F1	F2	F3	F4	F5	F6
F1						
F2	0.335^**^					
F3	0.172	0.258^*^				
F4	0.526^**^	0.294^**^	0.236^*^			
F5	−0.079	−0.134	−0.048	0.050		
F6	0.275^*^	0.259^*^	0.381^**^	0.352^**^	0.010	

**Correlation is significant at the 0.01 level (2-tailed). *Correlation is significant at the 0.05 level (2-tailed).

Composite scores for each factor were derived by taking a weighted mean of the questionnaire items, with weights drawn directly from the pattern-matrix coefficients produced by the PAF with Promax rotation. For each respondent and each factor, we multiplied each item response by its corresponding loading on that factor and then summed these products, and then divided by the sum of the loadings. This procedure ensures that items contributing more strongly to a given latent dimension exert proportionally greater influence on the composite score, thereby aligning observed scores as closely as possible with the factor structure uncovered by the analysis. The resulting composites thus faithfully represent each student’s standing on the six empirically derived constructs.

### 4.1 Learning motivation

H1 proposed that students who perceive ChatGPT as a valuable tool for problem solving would also demonstrate stronger learning retention and skill acquisition. The Pearson correlation analysis revealed a significant positive relationship between *Perceived Utility for Problem Solving* (F1) and *Learning Retention/Skill Acquisition* (F2), *r*(81) = 0.34, *p* = 0.002. This finding indicates that students who recognize ChatGPT’s usefulness in addressing academic problems are more likely to report that they retain knowledge and acquire skills through its use. Thus, H1 was supported.

**Figure 2:**
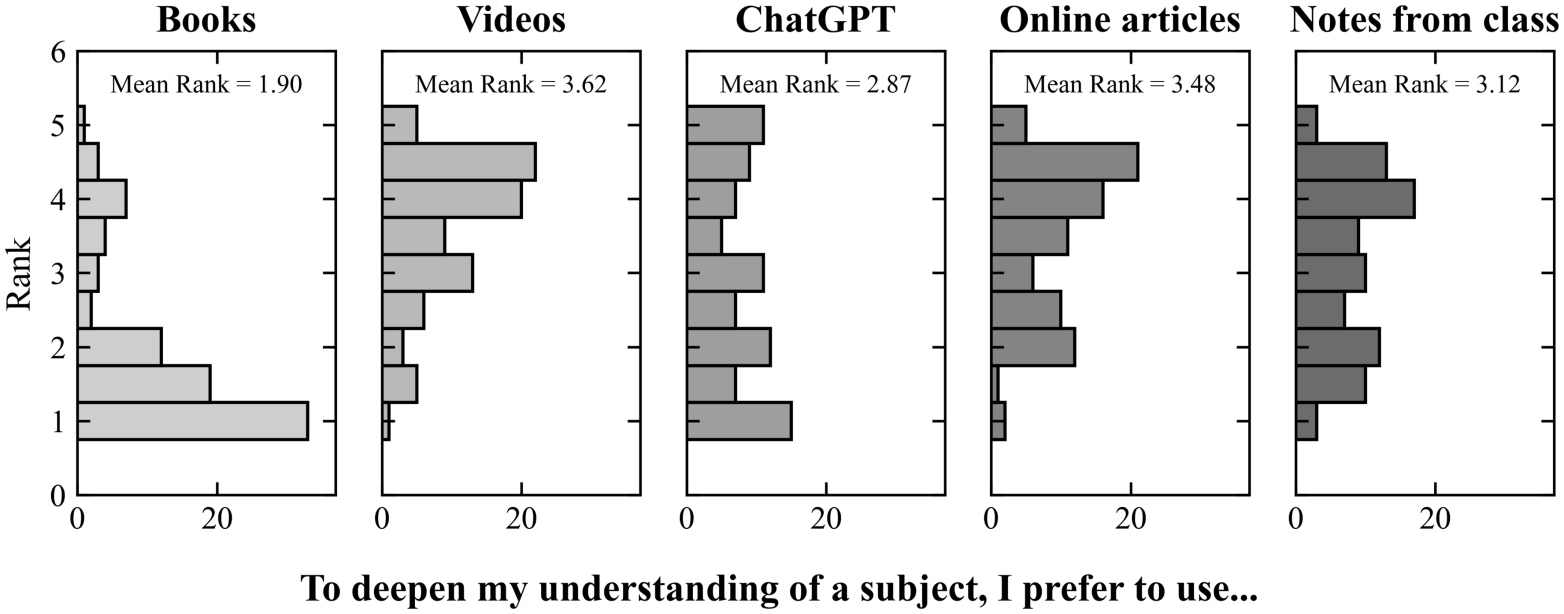
Friedman test results.

Beyond factor-level relationships, we also explored students’ broader learning preference. The participants were inquired about their preferred source for deepening their understanding of a subject and the results were quite interesting. Based upon these preferences, there are three groups that show up: (i) videos/video courses, online articles, and notes from professors; (ii) ChatGPT; (iii) Books. The above-mentioned list is ordered by interest in those sources. For books, the rank is lower than any other category (Books vs. all, pairwise post-hoc analysis after the Friedman test, all adjusted *p* ≤ 0.001). As depicted by Figure 2, interest in ChatGPT, ranked lower for all sources in the first group, with a statistically different result when compared to online articles (ChatGPT vs. Online Articles, pairwise post-hoc analysis after the Friedman test, adjusted *p* = 0.021). The students’ preference for ChatGPT over books and notes reflects a shift toward AI-driven learning, as noted by Chiu (2023).

The surveyed students express a clear preference for tangible assistance over theoretical guidance. Theoretical knowledge provides the intellectual scaffolding and foundational concepts critical for devising engineering solutions; however, theory alone is inadequate for comprehensive learning, as hands-on experience is equally vital. The practical application of theoretical principles serves to validate concepts while revealing their limitations. Engineers bridge the gap between theory and practice by applying academic knowledge to real-world challenges, adjusting as needed. Practical engagement fosters creativity, hones problem-solving skills, and deepens understanding of the complexities inherent in engineering (Allen, 2009; Reich et al., 2014). Regarding technical challenges, students report that ChatGPT enables them to arrive at effective solutions more swiftly and find it straightforward to replicate the steps when necessary. To a regular student, this help might seem like the road to autonomy (i.e., gaining skills), sustaining H1; however, does not necessarily reflect genuine skill acquisition.

The influence of ChatGPT on student motivation is significant and intricate. An exploratory study (Lai et al., 2023) employing structural equation modeling identified intrinsic motivation as the primary factor facilitating the acceptance of ChatGPT. The perceived usability of ChatGPT has significantly influenced student behavior. The study’s results underscore the necessity for ongoing enhancement of answer quality and user experience to fulfill the educational potential of chatbots. The respondents concur with the existence of the motivating stimuli, as indicated by the responses of I37 (Mdn = 4). This way, we conclude that the introduction of this component is warranted, as the chatbot assists students in comprehending the concepts or procedures fundamental to solving difficulties they once considered insurmountable.

### 4.2 AI literacy

H2 predicted that students who engage in more structured interactions with familiar topics would be more likely to verify the accuracy of ChatGPT-generated output. Results showed a significant positive correlation between *Structured Interaction with Known Topics* (F3) and *Verification Behavior* (F6), *r*(84) = 0.38, *p* < 0.001. To examine the predictive effect, a linear regression was conducted with F6 as the dependent variable and F3 as the predictor. The model was significant, *F* (1, 82) = 13.95, *p* < 0.001, accounting for 14.5% of the variance (*R^2^* = 0.15). Structured interaction was a significant predictor of verification behavior (*β* = 0.38, *p* < 0.001). These results provide support for H2, suggesting that students who structure their queries and interactions more carefully are also more conscientious about validating ChatGPT’s output.

Students’ preference for practical support resonates deeply with the application-driven ethos of engineering education (Allen, 2009; Reich et al., 2014), indicating that ChatGPT can meaningfully enhance firsthand learning experiences. Yet, the risk of over-reliance on such tools sparks valid concerns about the erosion of critical thinking skills, as cautioned by Thaker and Thaker (2024) and Crawford et al. (2024). In contrast to Hosseini et al. (2023), our research distinctly illuminates the nuanced perspectives of technical students, filling a critical gap in studies centered on student experiences.

An initial study of the incoherent responses generated by ChatGPT reveals that students recognize a distinction among them. Their perspective indicates that the prevalence of logical errors in responses surpasses that of inaccurate facts. Hsu and Thompson (2023) asserted that AI models are susceptible to disseminating misinformation—yet some students remain oblivious to this reality or deliberately disregard it, sustaining H2 and raising ethical issues.

Analyzing the trends among engineering students, a Friedman test concluded that there is no statistical difference between the perceived level of usefulness when it comes to different motives for which Large Language Models are used, i.e., lack of time, understanding a concept, solving assignments, resolving technical issues, writing essays (Friedman test, $\chi^{2}\left(4\right)$ = 6.663, *p* = 0.155).

**Figure 3:**
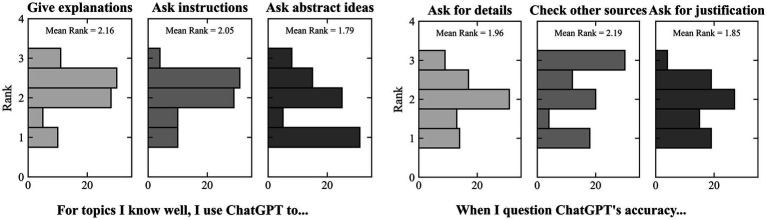
Friedman test results.

**Figure 4:**
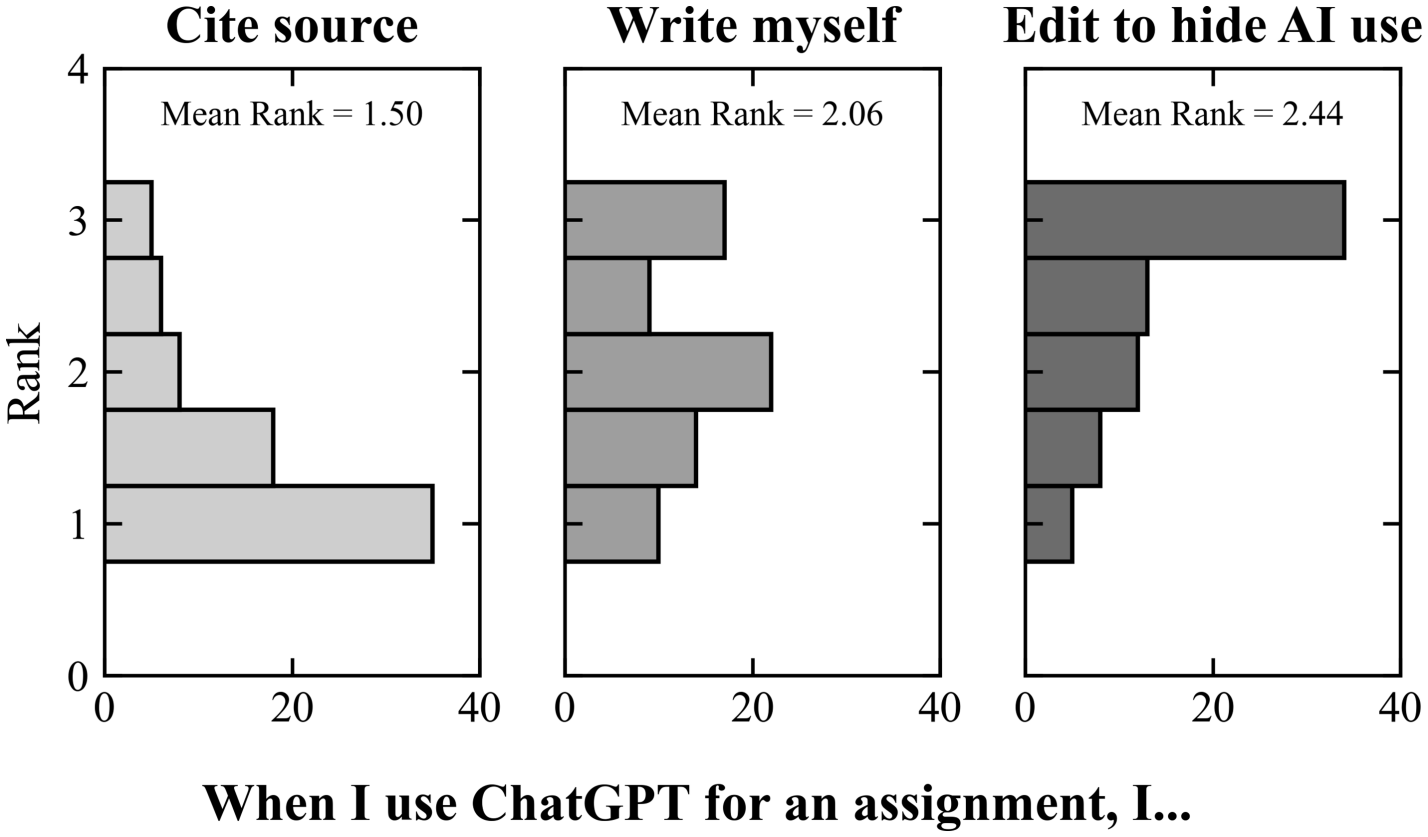
Friedman test result for I17-19.

The data revealed that while having a conversation with a ChatGPT bot, students tend to ask for exact instructions, have an eager attitude toward engaging in a dialog with the language model, and provide explanations, even in subjects students consider themselves proficient in. The significant Friedman test ( $\chi^{2}\left(2\right)$ = 10.745, *p* = 0.005, Figure 3) indicates that users rank the usage purposes differently, favoring explanations and instructions over abstract ideas. This shows that students are more aware of the true scope of AI models, fostering H2. However, the low effect size (Kendall’s W = 0.064) signals that this pattern is not strongly consistent across all users — suggesting considerable individual variation in usage behavior. When students are unsure of the correctness of the information output by the language model, the data shows that there is no statistical difference between the frequencies of verifying the information in other sources and asking ChatGPT to justify the information (I13-I14 pairwise comparison post-hoc analysis after Friedman test, adjusted *p* = 0.076, Figure 4).

Upon being asked to distinguish between logical errors and incorrect information, the individuals said that it is more common to find logical errors (i.e., incoherent flow of ideas) rather than pure incorrect information (Wilcoxon Signed-Rank Test, W = 569.5, *p* = 0.003). This difference, with a moderate effect size (*r* = −0.23), suggests a consistent perception that ChatGPT’s responses tend to falter more in reasoning structure than in factual content.

Beyond these insights, several additional considerations emerge. Students are increasingly familiar with the mechanics of artificial intelligence (AI), yet there remains a pressing need to deepen their understanding of its ethical and societal implications (Tzirides et al., 2024; Segbenya et al., 2024; Gouseti et al., 2024; Yim, 2024; Crawford et al., 2024; Salih et al., 2024). While they tend to employ AI as a supportive tool, there is a risk of developing over-reliance (Crawford et al., 2024; Salih et al., 2024). Students demonstrate growing awareness of AI’s ethical dimensions but require further education, particularly concerning issues of confidentiality, bias, and fairness (Ho et al., 2024; Nemorin, 2024; Gouseti et al., 2024). Furthermore, AI is widely utilized for research, data analysis, feedback, and assessment (Salih et al., 2024; Segbenya et al., 2024; Donmez, 2024). Students adopt a balanced perspective, valuing AI’s utility while remaining mindful of its limitations (Crawford et al., 2024; Salih et al., 2024; Segbenya et al., 2024).

H3 stated that students who engage more frequently in consulting unfamiliar topics and interacting with familiar ones in a structured way would report greater learning gains and increased reliance on ChatGPT. Correlation analyses indicated that both *Structured Interaction with Known Topics* (F3) and *Consultation on Unfamiliar Topics* (F4) were positively associated with *Learning Gains and Dependence* (F6) (F3: *r* = 0.38, *p* < 0.001; F4: *r* = 0.35, *p* = 0.001). A multiple regression analysis including F3 and F4 as predictors of F6 confirmed the hypothesis, yielding a significant model, *F*(2, 81) = 11.28, *p* < 0.001, with an explained variance of 21.8% (*R^2^* = 0.22). Both F3 (*β* = 0.32, *p* = 0.002) and F4 (*β* = 0.28, *p* = 0.008) were significant predictors. These findings support H3, suggesting that greater use of ChatGPT for both familiar and unfamiliar academic tasks is linked to stronger perceptions of learning but also reflects increased dependence on the tool.

### 4.3 Plagiarism

The attitudes toward the use of AI while solving assignments vary (Friedman test, $\chi^{2}\left(2\right)$ = 37.537, *p* < 0.001). Following the Friedman test with homogeneous subsets post-hoc analysis, we notice that all three questioned attitudes (citing the source, not using AI, and making subtle changes such that the use of AI is indistinguishable from human-written text) are found on three distinct levels, as shown in Figure 4. The students reported that changing subtle things in the output of language models is the “go-to” method when using AI for solving assignments, while rarely mentioning that the assignment contains AI-generated information. Obviously, this strategy is nothing but plagiarism at its finest, giving a direction toward H4. Remarkably, there is a medium level of interest in fully human-made assignments among students since the emergence of AI models (see Figure 4).

From our findings we infer that technical students view ChatGPT as an asset for academic tasks, consistent with observations by Salih et al. (2024) and Segbenya et al. (2024). The correlation between time constraints and the use of ChatGPT for assignments underscores its role as a time-efficient resource, aligning with Egunjobi and Adeyeye (2024). However, the strong association between effortless content access and the lack of proper citations raises concerns about plagiarism risks, echoing findings by Lee et al. (2024) and Nemorin (2024). This result partially contrasts with Costa et al. (2024), who suggest that AI literacy mitigates unethical conduct, a claim our data only slightly supports; students with greater AI literacy tend to verify AI-generated outputs.

It should be noted, however, that our exploratory factor analysis did not identify a distinct factor corresponding to plagiarism-related behaviors. As a result, we do not have a validated construct to directly test Hypothesis 4. While the observed patterns—such as students subtly modifying AI-generated text or rarely citing AI sources—suggest potential concerns regarding academic integrity, the current data does not allow for definitive claims. This limitation underscores the need for further research to develop specific measures of plagiarism in the context of AI-assisted academic work, while the present findings remain informative regarding students’ usage patterns, verification behaviors, and attitudes toward ChatGPT.

## 5 Conclusion

This research provides a balanced view of students’ educational use of generative AI models, specifically ChatGPT, through the identification of six underlying factors driving their patterns of use. These categories—from practical usefulness to moral uncertainty—constitute a cognitive–behavioral lens through which students engage with AI-assisted learning.

### 5.1 Learning motivation

Consistent with Hypothesis 1, students who perceived ChatGPT as a helpful tool for problem solving also reported greater learning retention and skill acquisition. This finding highlights that when learners view ChatGPT as a support for clarifying concepts and solving tasks, they are more likely to attribute actual learning outcomes to their use. In line with prior research on educational technology adoption, positive utility perceptions appear to reinforce motivation and engagement with material, leading to self-reported learning benefits.

### 5.2 AI literacy and verification

Hypothesis 2 was also supported, showing that structured interactions with familiar topics significantly predicted verification behaviors. Students who approached ChatGPT with more deliberate and organized queries were also more likely to critically evaluate its responses. This relationship reflects a form of AI literacy: rather than passively accepting generated output, these students engaged in practices of verification, which aligns with ethical and responsible AI use. Importantly, this suggests that pedagogical efforts to encourage structured prompting may also enhance students’ ability to verify information quality.

### 5.3 AI literacy and dependence

In support of Hypothesis 3, both structured interactions with familiar topics and consultations on unfamiliar topics were positively associated with perceived learning gains and dependence on ChatGPT. While this indicates that students recognize value in using the tool across diverse contexts, it also raises concerns about potential over-reliance. The finding that increased exploration correlates with greater dependence suggests a tension between the benefits of expanded learning opportunities and the risk of diminishing autonomous problem-solving skills.

### 5.4 Plagiarism risk

No factor representing plagiarism emerged from the exploratory analysis, preventing a formal test of Hypothesis 4. While students reported modifying AI-generated text or rarely citing ChatGPT, these observations are indicative rather than conclusive. Future research should develop specific measures to capture plagiarism behaviors in AI-assisted academic work.

Consequently, the integration of AI into academic life offers significant benefits when approached with careful consideration of its implications and influencing factors. The three dimensions—learning motivation, AI literacy, and plagiarism & ethics—must be addressed holistically to avoid misuse and unintended consequences in AI application. We endorse AI as a learning partner, provided it does not impair cognitive functions, foster dependency, or operate outside a well-defined ethical framework. Dependency may limit critical thinking and inclusive perspectives, while using technology without integrity is a dangerous path. Ethical principles must be formally established, practiced, and internalized. These findings prompt further reflection: does unrestricted AI access encourage ethical and responsible use, or does it fuel a desire for power and false competence? Since AI is created by humans with specific values and knowledge, its algorithms reflect those biases—can we connect students to a narrow set of values without exposing them to humanity’s broader spectrum? Does unfettered AI access inadvertently restrict users to certain values, marginalizing others?

Therefore, to ensure the healthy and progressive integration of artificial intelligence (AI) into society, we need precise foresight regarding the factors that shape its responsible use, enabling rapid adaptation, sound decision-making in novel situations, and the cultivation of ethical behaviors. This approach not only harnesses AI’s potential to drive human progress but also safeguards against unintended consequences, fostering a future where technology enhances our collective growth while preserving integrity and accountability.

In short, this study demonstrates that although students perceive ChatGPT as an engaging and motivating learning tool, its educational potential is conditioned by the way—and the reasons why—it is actually utilized. The data highlights the imperatives of addressing learning motivation, AI literacy, and academic integrity as interdependent facets of any agenda for responsible AI adoption in education. It is not enough to advance technical proficiency alone; students must also be provided with systematic guidance on AI learning’s ethical and epistemological limits.

### 5.5 Limitations

Our statistical findings stem from a convenience-based sample, which may not fully capture the experiences of students worldwide. The sample, drawn exclusively from a technical university, limits the applicability of the results to broader contexts. Focusing solely on STEM disciplines, the study may not reflect the realities of fields like medicine, arts, or humanities. Additionally, the absence of a qualitative research component narrows the depth of our insights. The research relied on a structured questionnaire, with self-reported data that could be influenced by biases. Due to uneven sample sizes between undergraduate and master’s students, comparative analysis was not feasible, further constraining our conclusions. However, the fact that 70% of respondents were first-year students suggests that early exposure to AI tools significantly shapes their perspectives. Further studies should prioritize larger, more diverse samples and longitudinal approaches to better understand the long-term effects of AI use in education.

## 6 Pedagogical recommendations

Drawing on our research and the insight from Guan et al. (2024) that educators must adapt to new roles in AI-driven education, focusing on guidance and support rather than merely delivering information, we propose the following strategies to responsibly integrate ChatGPT into educational settings:

Educators should engage in professional development to deepen their understanding of AI tools (Sharples, 2023; Giannakos et al., 2024; Halaweh, 2023; Hodges and Ocak, 2023). This training would empower them to guide students in leveraging ChatGPT effectively for learning while proactively addressing risks such as plagiarism, as highlighted by Salih et al. (2024). Embedding this guidance within the learning process fosters a balanced and ethical approach to AI use.

Institutions should prioritize the development of clear, comprehensive guidelines for AI use, with a strong emphasis on proper citation practices to uphold academic integrity, as underscored by Lee et al. (2024). A critical first step is crafting a university-wide strategy for integrating AI into the learning process. This session should be followed by the adoption of a code of ethics for AI use, collaboratively agreed upon by all stakeholders—students, educators, and administrators. These ethical guidelines, rooted in principles of fairness and accountability, would inform the creation of practical usage protocols, ensuring a cohesive framework.

AI literacy should be woven into technical curricula, equipping students with the skills to critically evaluate AI-generated outputs (Tzirides et al., 2024; Costa et al., 2024; Gidiotis and Hrastinki, 2024; Kalal et al., 2023; Mollick, 2023). This approach empowers students to use ChatGPT as a tool for learning while maintaining intellectual rigor and independence.

Finally, blending ChatGPT with traditional teaching methods can nurture critical thinking and social engagement (Liu and Yushchik, 2024; Mahapatra, 2024; Nye et al., 2014; Padhiyar and Modha, 2024; Pérez-Marín et al., 2006; Popovici, 2023; Yang, 2023). By combining AI’s capabilities with interactive, human-centered pedagogies, educators can ensure that ChatGPT enhances, rather than overshadows, holistic learning experiences.

## Data Availability

The datasets presented in this study can be found in online repositories. The names of the repository/repositories and accession number(s) can be found at: https://osf.io/e35mq/.

## References

[ref1] Allen J. (2009). Valuing practice over theory: how beginning teachers re-orient their practice in the transition from the university to the workplace. Teach. Teach. Educ. 25, 647–654. doi: 10.1016/j.tate.2008.11.011

[ref2] Ayman S. E. El-Seoud S. A. Nagaty K. Karam O. (2023). *The impact of ChatGPT on student learning and performance*. ResearchGate.

[ref3] Chiu T. K. F. (2023). The impact of generative AI (GenAI) on practices, policies, and research direction in education: a case of ChatGPT and Midjourney. Interact. Learn. Environ. 32, 6187–6203. doi: 10.1080/10494820.2023.2253861

[ref4] Costa A. R. Lima N. Viegas C. Caldeira A. (2024). Critical minds: enhancing education with ChatGPT. Cogent Educ. 11:286. doi: 10.1080/2331186X.2024.2415286

[ref5] Crawford J. Allen K. A. Pani B. Cowling M. (2024). When artificial intelligence substitutes humans in higher education: the cost of loneliness, student success, and retention. Stud. High. Educ. 49, 883–897. doi: 10.1080/03075079.2024.2326956

[ref6] Dancey C. P. Reidy J. (2004). Statistics without maths for psychology. Hoboken, NJ: Pearson Prentice Hall.

[ref9001] Davis F. D. (1989). Perceived usefulness, perceived ease of use, and user acceptance of information technology. MIS Quarterly. 13, 319–340. doi: 10.2307/249008

[ref7] Donmez M. (2024). Ai-based feedback tools in education: a comprehensive bibliometric analysis study. Int. J. Assess. Tools Educ. 11, 622–646. doi: 10.21449/ijate.1467476

[ref8] Egunjobi D. Adeyeye O. J. (2024). Revolutionizing learning: the impact of augmented reality (AR) and artificial intelligence (AI) on education. Int. J. Res. Publ. Rev. 5, 1157–1170. doi: 10.55248/gengpi.5.1024.2734

[ref9] Escalante J. Pack A. Barrett A. (2023). Ai-generated feedback on writing: insights into efficacy and ENL student preference. Int. J. Educ. Technol. High. Educ. 20:425. doi: 10.1186/s41239-023-00425-2

[ref10] Gambo I. Abegunde F. J. Gambo O. Ogundokun R. O. Babatunde A. N. Lee C. C. (2024). Grad-AI: an automated grading tool for code assessment and feedback in programming course. Educ. Inf. Technol. 30, 9859–9899. doi: 10.1007/s10639-024-13218-5

[ref11] Giannakos M. Azevedo R. Brusilovsky P. Cukurova M. Dimitriadis Y. Hernandez-Leo D.. (2024). The promise and challenges of generative AI in education. Behav. Inf. Technol. 44, 2518–2544. doi: 10.1080/0144929X.2024.2394886

[ref12] Gidiotis I. Hrastinski S. (2024). Imagining the future of artificial intelligence in education: a review of social science fiction. Learn. Media Technol. 2024:829. doi: 10.1080/17439884.2024.2365829

[ref13] Gouseti A. James F. Fallin L. Burden K. (2024). The ethics of using AI in K-12 education: a systematic literature review. Technol. Pedagogy Educ. 34, 161–182. doi: 10.1080/1475939X.2024.2428601

[ref14] Grájeda A. Burgos J. Córdova P. Sanjinés A. (2023). Assessing student-perceived impact of using artificial intelligence tools: construction of a synthetic index of application in higher education. Cogent Educ. 11:917. doi: 10.1080/2331186X.2023.2287917

[ref15] Guan L. Zhang Y. Gu M. M. (2024). Pre-service teachers preparedness for AI-integrated education: an investigation from perceptions, capabilities, and teachers’ identity changes. Comput. Educ. 8:341. doi: 10.1016/j.caeai.2024.100341

[ref16] Halaweh M. (2023). ChatGPT in education: strategies for responsible implementation. Contemp. Educ. Technol. 15:ep421. doi: 10.30935/cedtech/13036

[ref17] Heeg D. M. Avraamidou L. (2023). The use of artificial intelligence in school science: a systematic literature review. Educ. Media Int. 60, 125–150. doi: 10.1080/09523987.2023.2264990

[ref18] Ho M. T. Mantello P. Vuong Q. H. (2024). Emotional AI in education and toys: investigating moral risk awareness in the acceptance of AI technologies from a cross-sectional survey of the Japanese population. Heliyon 10:e36251. doi: 10.1016/j.heliyon.2024.e36251, PMID: 39253209 PMC11382058

[ref19] Hodges C. B. Ocak C. (2023). *Integrating generative AI into higher education: Considerations*. EDUCAUSE Review. Available online at: https://er.educause.edu/articles/2023/8/integrating-generative-ai-into-higher-education-considerations.

[ref20] Hosseini M. Gao C. A. Liebovitz D. Carvalho A. M. Ahmad F. S. Luo Y.. (2023). An exploratory survey about using ChatGPT in education, healthcare, and research. PLoS One 18:Article e0292216. doi: 10.1371/journal.pone.0292216 37796786 PMC10553335

[ref21] Hsu T. Thompson S. A. (2023). *Disinformation researchers raise alarms about a.I. Chatbots*. The New York Times. Available online at: https://www.nytimes.com/2023/02/08/technology/ai-chatbots-disinformation.html.

[ref22] Huang S. Lai X. Ke L. Li Y. Wang H. Zhao X.. (2024). AI technology panic—is AI dependence bad for mental health? A cross-lagged panel model and the mediating roles of motivations for AI use among adolescents. Psychol. Res. Behav. Manag. 17, 1087–1102. doi: 10.2147/PRBM.S440889, PMID: 38495087 PMC10944174

[ref23] Hwang G. J. Xie H. Wah B. W. Gašević D. (2020). Vision, challenges, roles, and research issues of artificial intelligence in education. Comput. Educ. 1:100001. doi: 10.1016/j.caeai.2020.100001, PMID: 40930946

[ref24] Jansen T. Höft L. Bahr L. Fleckenstein J. Möller J. Köller O.. (2024). Comparing generative AI and expert feedback to students’ writing: insights from student teachers. Psychol. Erzieh. Unterr. 1, 80–92. doi: 10.2378/peu2024.art08d

[ref25] Kalal P. Pradhan M. S. Nikhil N. Vijay V. (2023). *Effect of ChatGPT on students’ creativity from academician’s and students’ perspective*. ResearchGate. Available online at: https://www.researchgate.net/publication/371912289_EFFECTOF_CHATGPT_ON_STUDENTS_CREATIVITY_FROM_ACADEMICIAN'S_AND_STUDENTS_PERSPECTIVE.

[ref26] Koltovskaia S. (2020). Student engagement with automated written corrective feedback (AWCF) provided by Grammarly: a multiple case study. Assess. Writing 44:450. doi: 10.1016/j.asw.2020.100450

[ref27] Lai C. Y. Cheung K. Y. Seng C. C. (2023). Exploring the role of intrinsic motivation in ChatGPT adoption to support active learning: an extension of the technology acceptance model. Comput. Educ. 5:Article 100178. doi: 10.1016/j.caeai.2023.100178

[ref28] Lee D. Arnold M. Srivastava A. Plastow K. Strelan P. Ploeckl F.. (2024). The impact of generative AI on higher education learning and teaching: a study of educators’ perspectives. Comput. Educ. 6:100221. doi: 10.1016/j.caeai.2024.100221, PMID: 40930946

[ref29] Liu Z. Y. Yushchik E. (2024). Exploring the prospects of using artificial intelligence in education. Cogent Educ. 11:464. doi: 10.1080/2331186X.2024.2353464

[ref30] Mahapatra S. (2024). Impact of ChatGPT on ESL students’ academic writing skills: a mixed methods intervention study. Smart Learn. Environ. 11:295. doi: 10.1186/s40561-024-00295-9

[ref31] Maphosa V. Maphosa M. (2023). Artificial intelligence in higher education: a bibliometric analysis and topic modeling approach. Appl. Artif. Intell. 37:1730. doi: 10.1080/08839514.2023.2261730

[ref32] Mollick E. (2023). *All my classes suddenly became AI classes*. One Useful Thing. Available online at: https://oneusefulthing.substack.com/p/all-my-classes-suddenly-became-ai.

[ref33] Morales-García W. C. Sairitupa-Sanchez L. Z. Morales-García S. B. Morales-García M. (2024). Development and validation of a scale for dependence on artificial intelligence in university students. Front. Educ. 9:1323898. doi: 10.3389/feduc.2024.1323898

[ref34] Nemorin S. (2024). Towards decolonising the ethics of AI in education. Global. Soc. Educ. 2024:3821. doi: 10.1080/14767724.2024.2333821

[ref35] Norušis M. J. (2011). IBM SPSS statistics 19 guide to data analysis. 1st Edn. Boston, MA: Addison Wesley.

[ref36] Nye B. D. Graesser A. C. Hu X. (2014). Autotutor and family: a review of 17 years of natural language tutoring. Int. J. Artif. Intell. Educ. 24, 427–469. doi: 10.1007/s40593-014-0029-5

[ref37] Padhiyar R. Modha S. (2024). Impact of the usage of ChatGPT on creativity among postgraduate students. Int. J. Sustain. Soc. Sci. 2, 83–92. doi: 10.59890/ijsss.v2i1.1376

[ref38] Parker B. D. (2024). Considering the impact of AI on the professional status of teaching. The Clearing House: J. Educ. Strateg. Issues Ideas 97, 233–236. doi: 10.1080/00098655.2024.2441805, PMID: 40922668

[ref39] Pérez-Marín D. Alfonseca E. Rodríguez P. (2006). “On the dynamic adaptation of computer assisted assessment of free-text answers” in Lecture notes in computer science. ed. Pérez-Marín D. (Berlin: Springer), 374–377.

[ref40] Polit D. F. Beck C. T. (2006, 2006). The content validity index: are you sure you know what’s being reported? Critique and recommendations. Res. Nurs. Health 29, 489–497. doi: 10.1002/nur.20147, PMID: 16977646

[ref41] Polit D. F. Beck C. T. Owen S. V. (2007). (2007). Is the CVI an acceptable indicator of content validity? Appraisal and recommendations. Res. Nurs. Health 30, 459–467. doi: 10.1002/nur.20199, PMID: 17654487

[ref42] Popovici M. (2023). ChatGPT in the classroom: exploring its potential and limitations in a functional programming course. Int. J. Hum. Comput. Interact. 40, 7743–7754. doi: 10.1080/10447318.2023.2269006

[ref43] Pradana M. Elisa H. P. Syarifuddin S. (2023). Discussing chatgpt in education: a literature review and bibliometric analysis. Cogent Educ. 10:134. doi: 10.1080/2331186X.2023.2243134

[ref44] Qian Y. Wang J. Cai Y. (2023). Revolutionizing educational landscapes: a systematic review of Metaverse applications, paradigms, and emerging technologies. Cogent Educ. 10:4006. doi: 10.1080/2331186X.2023.2264006

[ref45] Rahiman H. U. Kodikal R. (2024). Revolutionizing education: artificial intelligence empowered learning in higher education. Cogent Educ. 11:3431. doi: 10.1080/2331186X.2023.2293431

[ref46] Reich A. Rooney D. Gardner A. Willey K. Boud D. Fitzgerald T. (2014). Engineers’ professional learning: a practice-theory perspective. Eur. J. Eng. Educ. 40, 366–379. doi: 10.1080/03043797.2014.967181

[ref90001] Ryan R. M. Deci E. L. (2000). Self-determination theory and the facilitation of intrinsic motivation, social development, and well-being. Am. Psychol. 55, 68–78. doi: 10.1037/0003-066X.55.1.68 11392867

[ref47] Salih S. Husain O. Hamdan M. Abdelsalam S. Elshafie H. Motwakel A. (2024). Transforming education with AI: a systematic review of ChatGPT’s role in learning, academic practices, and institutional adoption. Results Eng.:103837. doi: 10.1016/j.rineng.2024.103837

[ref48] Saraswat D. (2024). Ai-driven pedagogies: enhancing student engagement and learning outcomes in higher education. Int. J. Sci. Res. 13, 1152–1154. doi: 10.21275/SR241119221041

[ref49] Segbenya M. Senyametor F. Aheto S. P. K. Agormedah E. K. Nkrumah K. Kaedebi-Donkor R. (2024). Modelling the influence of antecedents of artificial intelligence on academic productivity in higher education: a mixed method approach. Cogent Educ. 11:7943. doi: 10.1080/2331186X.2024.2387943

[ref50] Sharples M. (2023). Towards social generative AI for education: theory, practices, and ethics. Learn.: Res. Pract. 9, 159–167. doi: 10.1080/23735082.2023.2261131, PMID: 40922668

[ref51] Shi H. Aryadoust V. (2024). A systematic review of AI-based automated written feedback research. ReCALL 36, 187–209. doi: 10.1017/S0958344023000265

[ref52] Skowronek M. Gilberti R. M. Petro M. Sancomb C. Maddern S. Jankovic J. (2021). Inclusive STEAM education in diverse disciplines of sustainable energy and AI. Energy and AI 7:124. doi: 10.1016/j.egyai.2021.100124

[ref53] Thaker R. Thaker R. K. (2024). Advancing reinforcement learning: the role of explainability, human, and AI feedback integration. Robot. Autom. Eng. J. 6:681. doi: 10.19080/RAEJ.2024.06.555681

[ref54] Tzirides A. O. Zapata G. Kastania N. P. Saini A. K. Castro V. Ismael S. A.. (2024). Combining human and artificial intelligence for enhanced AI literacy in higher education. Comput. Educ. Open 6:100184. doi: 10.1016/j.caeo.2024.100184

[ref55] Yang M. (2023). *New York City schools ban AI chatbot that writes essays and answers prompts*. The Guardian. Available online at: https://www.theguardian.com/us-news/2023/jan/06/new-york-city-schools-ban-ai-chatbot-chatgpt.

[ref56] Yang H. Gao C. Shen H. Z. (2023). Learner interaction with, and response to, AI-programmed automated writing evaluation feedback in EFL writing: an exploratory study. Educ. Inf. Technol. 29, 3837–3858. doi: 10.1007/s10639-023-11991-3

[ref57] Yao N. Wang Q. (2024). Factors influencing pre-service special education teachers’ intention toward AI in education: digital literacy, teacher self-efficacy, perceived ease of use, and perceived usefulness. Heliyon 10:e34894. doi: 10.1016/j.heliyon.2024.e34894, PMID: 39149079 PMC11325385

[ref58] Yim I. H. Y. (2024). A critical review of teaching and learning artificial intelligence (AI) literacy: developing an intelligence-based AI literacy framework for primary school education. Comput. Educ. 7:319. doi: 10.1016/j.caeai.2024.100319

[ref59] Yusoff M. S. B. (2019). ABC of content validation and content validity index calculation. Educ. Med. J. 11, 49–54. doi: 10.21315/eimj2019.11.2.6

[ref60] Zaman B. U. (2023). *Transforming education through AI benefits risks and ethical considerations*. ResearchGate. Available online at: https://www.researchgate.net/publication/374373743.

[ref61] Zhai C. Wibowo S. Li L. D. (2024). The effects of over-reliance on AI dialogue systems on students’ cognitive abilities: a systematic review. Smart Learn. Environ. 11, 1–20. doi: 10.1186/s40561-024-00316-7

[ref62] Zhang L. Xu J. (2024). The paradox of self-efficacy and technological dependence: unraveling generative AI’S impact on university students’ task completion. Internet High. Educ. 2024:978. doi: 10.1016/j.iheduc.2024.100978

[ref63] Zhang S. Zhao X. Zhou T. Kim J. H. (2024). Do you have AI dependency? The roles of academic self-efficacy, academic stress, and performance expectations on problematic AI usage behavior. Int. J. Educ. Technol. High. Educ. 21:34. doi: 10.1186/s41239-024-00467-0

