# Supplementary material for: The perceived impact of artificial intelligence on academic learning
Source: Front Artif Intell. 2025 Oct 3;8:1611183. doi: 10.3389/frai.2025.1611183 (PMC12531233; doi:10.3389/frai.2025.1611183)
Supplement: Supplementary file 1 [file Data_Sheet_1.docx]

***Addendum one. Questionnaire Items Recoding***

| **Code** | **Item** | **Construct** |
| --- | --- | --- |
| **I1** | I felt that ChatGPT was helpful for university when... [I was short on time]. | **Learning Motivation** |
| **I2** | I felt that ChatGPT was helpful for university when... [I was trying to understand a concept]. | **Learning Motivation** |
| **I3** | I felt that ChatGPT was helpful for university when... [I was doing homework]. | **Learning Motivation** |
| **I4** | I felt that ChatGPT was helpful for university when... [I was looking for solutions to a technical problem]. | **Learning Motivation** |
| **I5** | I felt that ChatGPT was helpful for university when... [I had to write a text]. | **Learning Motivation** |
| **I6** | When I consult ChatGPT about a topic I know well... [I engage in dialogue, provide detailed explanations]. | **AI Literacy** |
| **I7** | When I consult ChatGPT about a topic I know well... [I ask for precise instructions]. | **AI Literacy** |
| **I8** | When I consult ChatGPT about a topic I know well... [I ask for ideas, ask abstract questions]. | **AI Literacy** |
| **I9** | I often discover that ChatGPT's answers contain... [Incorrect information]. | **AI Literacy** |
| **I10** | I often discover that ChatGPT's answers contain... [Logical errors]. | **AI Literacy** |
| **I11** | I consult ChatGPT about topics I know well. | **AI Literacy** |
| **I12** | When I am unsure about the accuracy of a response from ChatGPT... [I ask it to explain in detail]. | **AI Literacy** |
| **I13** | When I am unsure about the accuracy of a response from ChatGPT... [I verify the information from other sources]. | **AI Literacy** |
| **I14** | When I am unsure about the accuracy of a response from ChatGPT... [I ask it to justify its choice]. | **AI Literacy** |
| **I15** | When I get help from ChatGPT with a problem, I better understand the steps and can solve a similar problem on my own. | **Learning Motivation** |
| **I16** | I view ChatGPT’s answers with skepticism because they can be wrong. | **AI Literacy** |
| **I17** | If I want to include content written by ChatGPT in an assignment... (answer if applicable) [I cite the source]. | **Plagiarism** |
| **I18** | If I want to include content written by ChatGPT in an assignment... (answer if applicable) [I prefer to write everything myself]. | **Plagiarism** |
| **I19** | If I want to include content written by ChatGPT in an assignment... (answer if applicable) [I make subtle changes, so it is not noticeable]. | **Plagiarism** |
| **I20** | When ChatGPT gives me instructions, I try them out to see if they work. | **Learning Motivation** |
| **I21** | By completing university assignments with the help of ChatGPT, I gained skills that stayed with me. (answer if applicable) | **Learning Motivation** |
| **I22** | Because I asked ChatGPT for explanations about a concept or process (not a direct solution), I understood how to solve a problem I otherwise did not know how to approach. | **Learning Motivation** |
| **I23** | When I have a problem I do not know how to solve, I often turn to ChatGPT. | **Learning Motivation** |
| **I24** | I usually consult ChatGPT about topics I am not familiar with. | **Learning Motivation** |
| **I25** | To deepen my understanding of a topic, I prefer to use... [Books]. | **Learning Motivation** |
| **I26** | To deepen my understanding of a topic, I prefer to use... [Videos / video courses]. | **Learning Motivation** |
| **I27** | To deepen my understanding of a topic, I prefer to use... [ChatGPT]. | **Learning Motivation** |
| **I28** | To deepen my understanding of a topic, I prefer to use... [Online articles]. | **Learning Motivation** |
| **I29** | To deepen my understanding of a topic, I prefer to use... [Lecture notes from professors]. | **Learning Motivation** |
| **I30** | When I ask ChatGPT for help with a technical problem... [I reach a solution much faster]. | **Learning Motivation** |
| **I31** | When I ask ChatGPT for help with a technical problem... [If I face the same problem again, I can handle it on my own]. | **Learning Motivation** |
| **I32** | It is still important for people to learn how to do certain tasks, even if ChatGPT can do them too (e.g., programming). | **AI Literacy** |
| **I33** | I often ask ChatGPT to summarize long texts so I can quickly grasp the main ideas. | **AI Literacy** |
| **I34** | I ask ChatGPT to explain articles or texts I do not fully understand. | **Learning Motivation** |
| **I35** | By default, ChatGPT uses too many words, which makes explanations hard to follow. | **Learning Motivation** |
| **I36** | I often ask ChatGPT to be concise in its explanations. | **Learning Motivation** |
| **I37** | When I ask ChatGPT for help with a problem, I feel encouraged, and it is easier to continue on my own. | **Learning Motivation** |
| **I38** | I often ask ChatGPT for precise technical information instead of checking a manual or using a search engine like Google. | **Plagiarism** |
